# Pulmonary Metastases From Undiagnosed Prostate Cancer Mimicking Late Recurrence of Renal Cell Carcinoma: Diagnosis Confirmed by Thoracoscopic Biopsy

**DOI:** 10.7759/cureus.103943

**Published:** 2026-02-19

**Authors:** Soma Tsuiki, Tomonari Oki, Shuhei Iizuka, Yoshiro Otsuki, Toru Nakamura

**Affiliations:** 1 Department of General Thoracic Surgery, Seirei Hamamatsu General Hospital, Hamamatsu, JPN; 2 Department of Pathology, Seirei Hamamatsu General Hospital, Hamamatsu City, JPN

**Keywords:** carcinoma, lung metastasis, renal cell, thoracic surgery, video-assisted

## Abstract

This case report concerns a 68-year-old man who presented with enlarging pulmonary nodules 24 years after undergoing a right radical nephrectomy for renal cell carcinoma (RCC). Given the long disease-free interval and the characteristic late recurrence pattern of RCC, pulmonary metastasis of renal origin was initially suspected. Considering the potential bleeding risk associated with the hypervascular nature of metastatic RCC, video-assisted thoracoscopic surgery (VATS) was performed for a definitive diagnosis. Histopathological analysis revealed a cribriform architecture, and immunohistochemical staining was positive for prostrate-specific antigen (PSA), NKX3.1, and alpha-methylacyl-CoA racemase (AMACR), leading to a diagnosis of metastatic prostate adenocarcinoma rather than a recurrence of RCC. Subsequent evaluation identified a primary prostate cancer (Gleason score 8, cT2cN0M1), and the patient achieved a favorable response with androgen deprivation therapy. This case highlights the pitfall of "diagnostic anchoring" to a previous malignancy and emphasizes that new pulmonary lesions, even decades after an initial cancer diagnosis, necessitate histological confirmation to differentiate between recurrence and a second primary malignancy. Video-assisted thoracoscopic surgery (VATS) remains a safe and effective diagnostic approach in such clinically ambiguous scenarios.

## Introduction

In patients with a history of malignancy who present with multiple pulmonary nodules, metastasis from the prior tumor is the primary consideration. However, when the disease-free interval is prolonged, other diseases - including a new primary malignancy or infectious etiologies - should also be considered. Therefore, histopathological confirmation is essential to avoid diagnostic anchoring and ensure an accurate diagnosis.

Renal cell carcinoma (RCC) recurs in approximately 20%-30% of patients even after radical nephrectomy, with the lungs being the most common site of metastasis [1,2]. RCC is also known for its potential late recurrence, with distant metastases reported even decades after initial treatment [3-5].

Because RCC is a hypervascular tumor that retains this feature at metastatic sites, risk of bleeding during biopsy is substantial [6,7]. Thus, when pulmonary metastasis from RCC is suspected, video-assisted thoracoscopic surgery (VATS) remains a valuable and safer alternative to transbronchial or percutaneous needle biopsy. We herein report a case in which multiple pulmonary nodules detected 24 years after nephrectomy for RCC were, upon VATS biopsy, diagnosed as metastases from previously undiagnosed prostate cancer.

## Case presentation

A 68-year-old man presented with progressively enlarging bilateral pulmonary nodules. Twenty-four years earlier, at the age of 44, he had undergone right radical nephrectomy for RCC.

**Figure 1:**
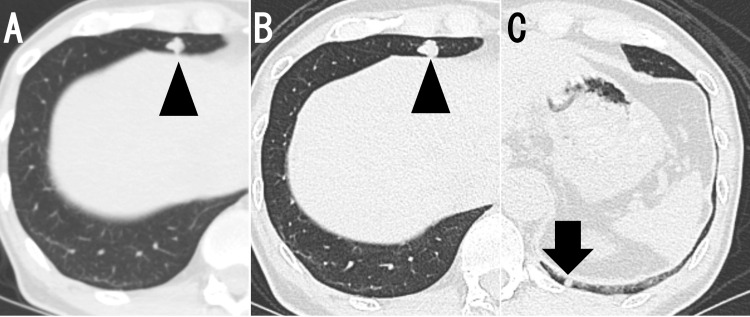
Chest CT A: Plain axial CT image showing a 10-mm nodule in the right middle lobe (arrowhead). B: The nodule enlarged to 12 mm four months later (arrowhead). C: Another nodule emerged in the left lower lobe (arrow).

The patient had been under regular follow-up with the gastroenterology department for suspected primary sclerosing cholangitis, during which a follow-up computed tomography (CT) scan, obtained four months prior to his current presentation, incidentally revealed an irregular 10-mm nodule in the right middle lobe of the lung (Figure 1A). Four months later, the nodule had increased in size to approximately 12 mm, and another nodule was newly identified in the left lower lobe. Given the patient’s history of RCC, pulmonary metastases were suspected, and VATS was planned for diagnostic purposes.

**Figure 2:**
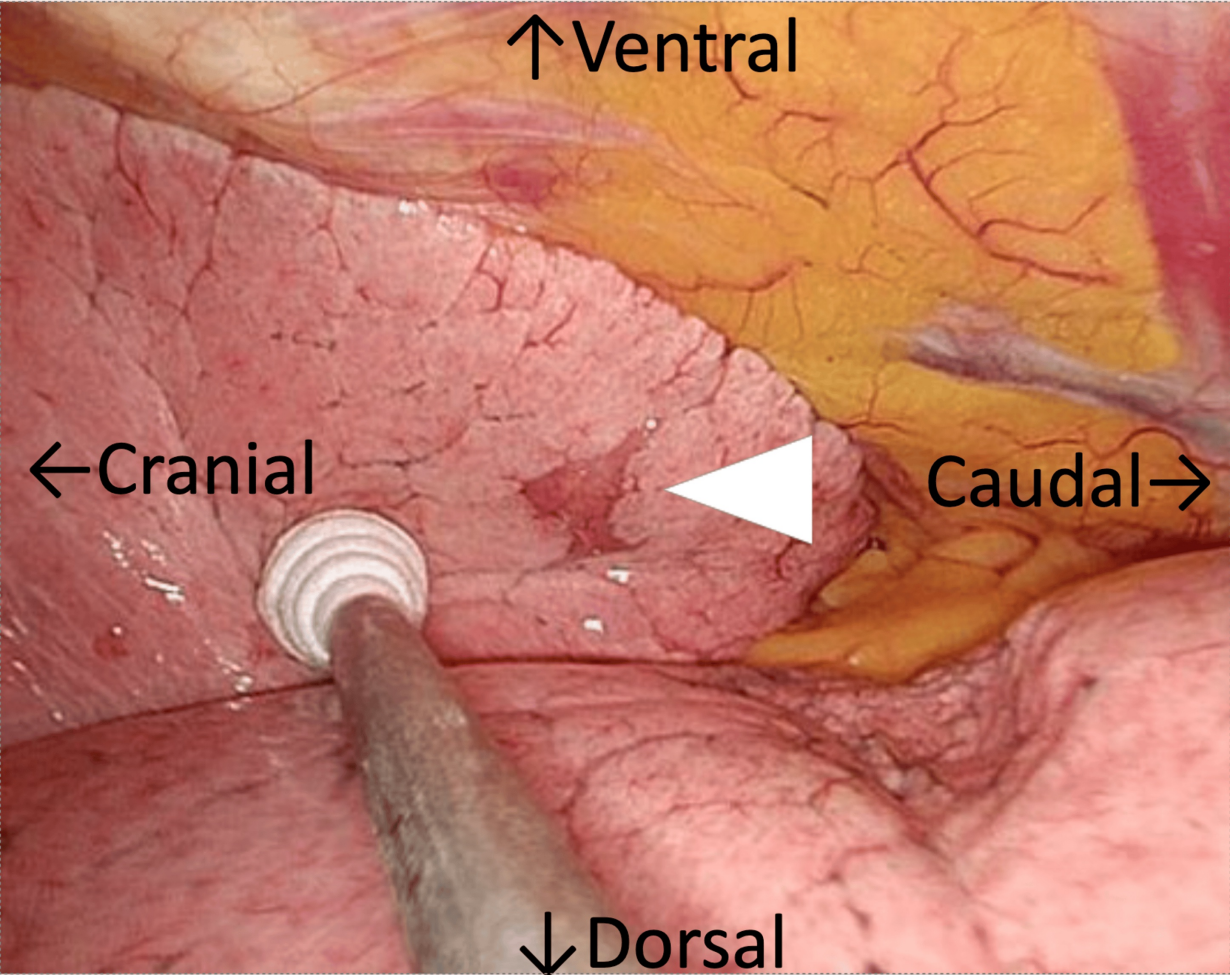
Operative view Thoracoscopy revealed a well-demarcated, pale reddish nodule approximately 1.5 cm in size (arrowhead).

The procedure was performed with the patient in the left lateral decubitus position under one-lung ventilation. Thoracoscopy revealed a well-demarcated, pale reddish nodule approximately 1.5 cm in diameter in segment 5 of the lung (Figure 2).

A wedge resection was performed, securing a 1 cm surgical margin.

**Figure 3:**
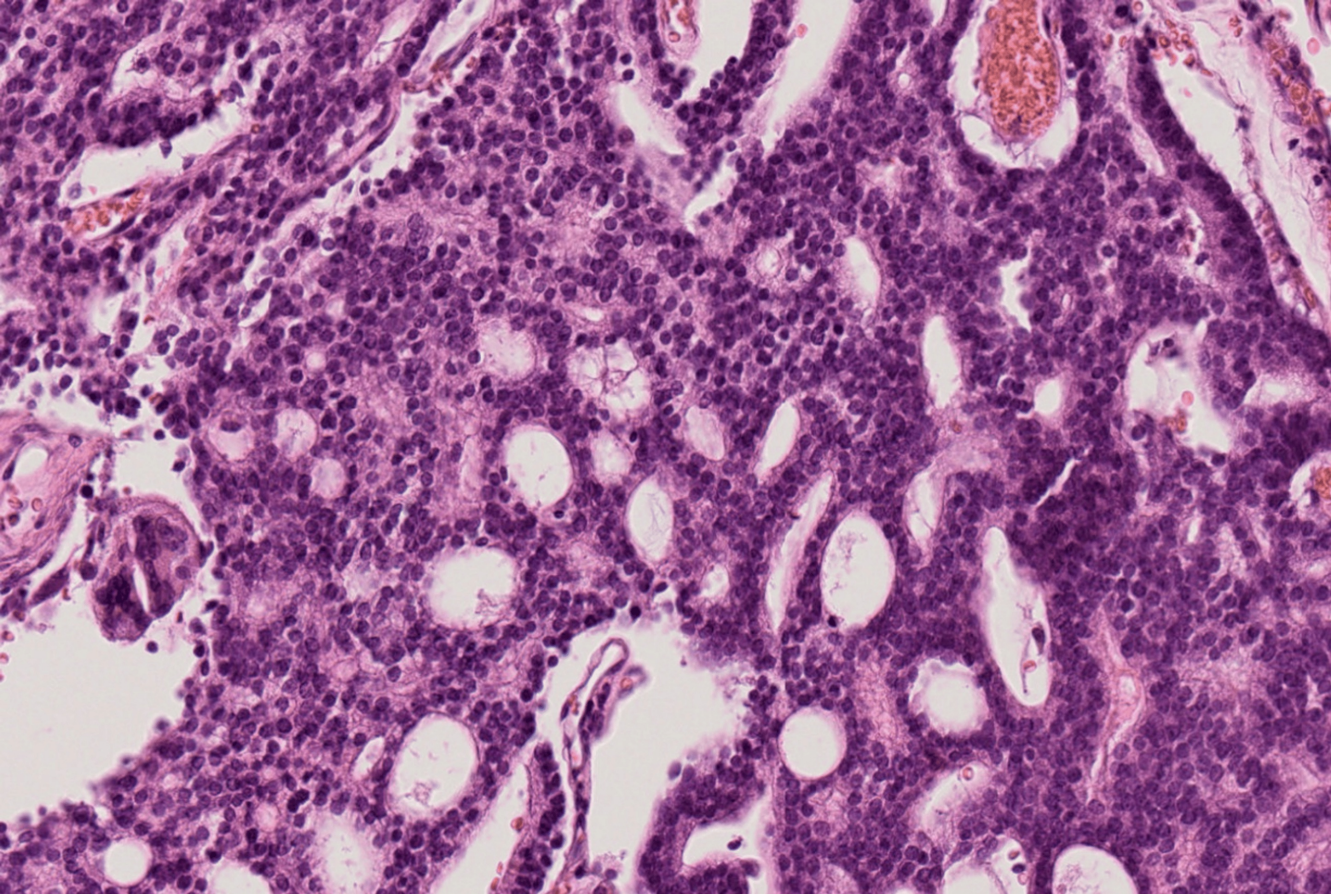
Hematoxylin-eosin staining Hematoxylin-eosin staining showing glandular structures composed of epithelial-like cells exhibiting a cribriform pattern (×20).

**Figure 4:**
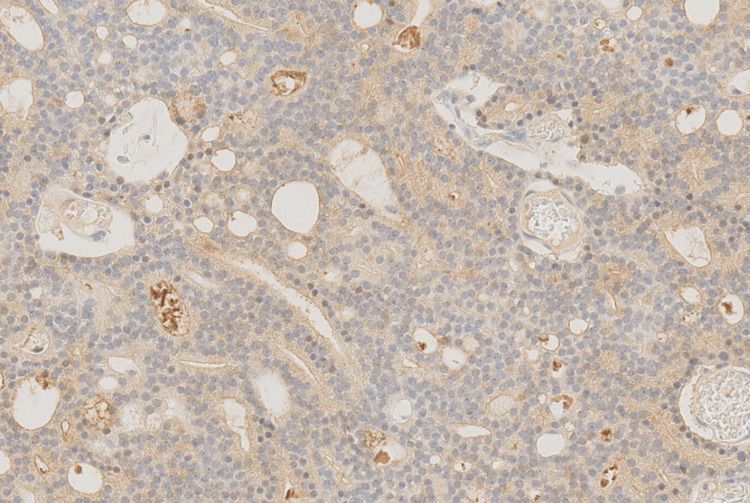
Positive PSA staining Positive prostate-specific antigen (PSA) staining, appearing as brown cytoplasmic staining within the cancer cells (×20).

**Figure 5:**
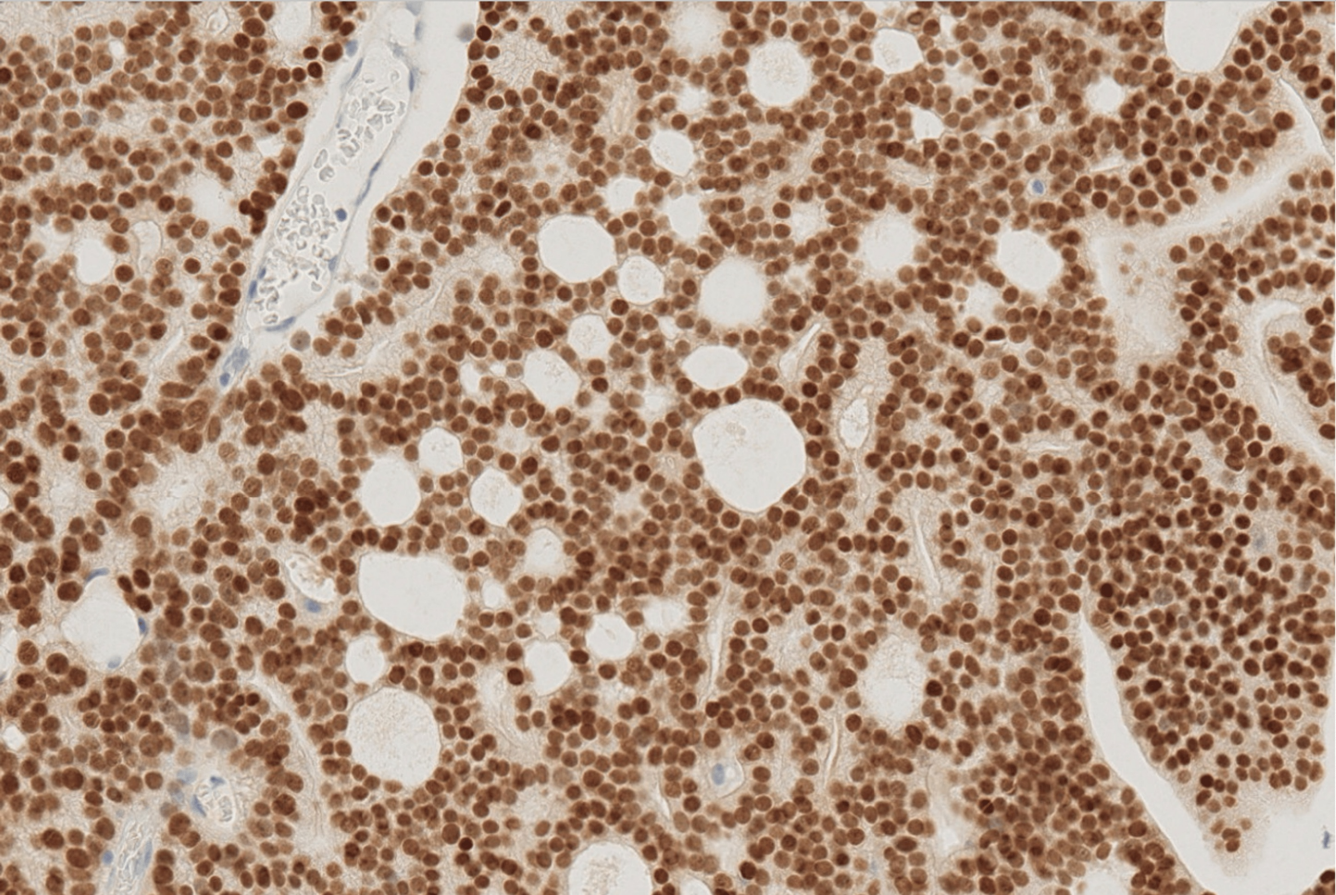
Positive NKX3.1 staining Positive NKX3.1 staining, characterized by brown nuclear staining in the cancer cells (×20).

**Figure 6:**
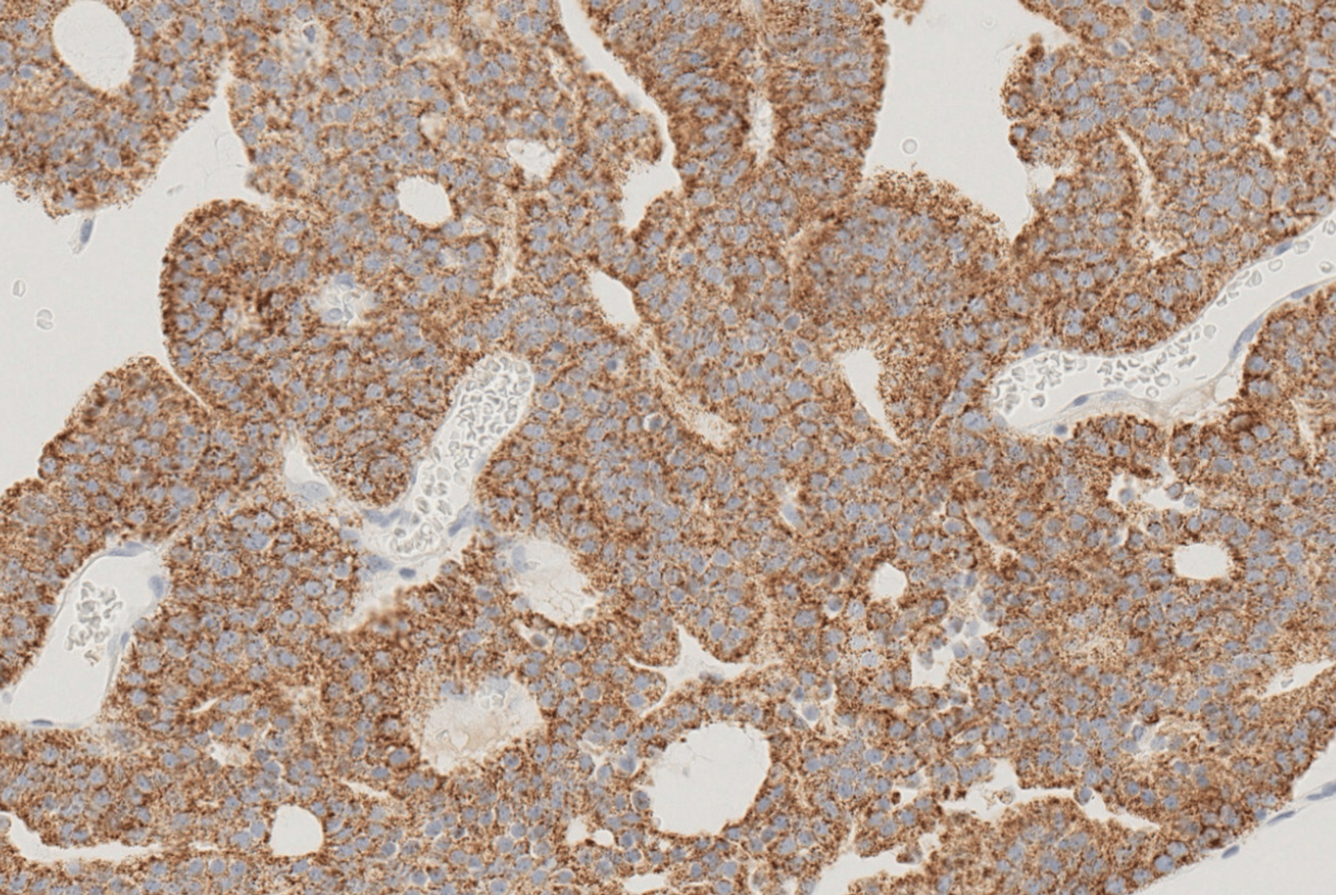
Positive AMACR staining Positive α-methylacyl-CoA racemase (AMACR) staining, showing brown cytoplasmic staining in the cancer cells (×20).

Histopathological examination revealed epithelial-like cells with a cribriform architecture. Immunohistochemical staining was positive for prostate-specific antigen (PSA), NKX3.1, and α-methylacyl-CoA racemase (AMACR), all of which are highly specific for prostate carcinoma (Figures 3-6).

**Figure 7:**
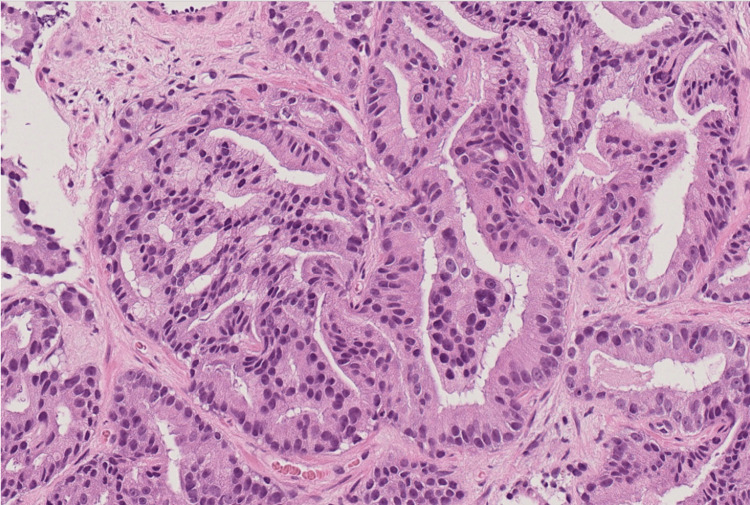
Hematoxylin-eosin staining of the biopsied specimen from the prostate. Hematoxylin-eosin staining showing adenocarcinoma in the prostate with a loss of normal glandular architecture (×20).

A subsequent transrectal ultrasound-guided prostate biopsy confirmed adenocarcinoma with a Gleason score of 8 (Figure 7). Cancer cells were identified in both lobes of the prostate. The patient was asymptomatic. Digital rectal examination revealed a stony-hard prostate, and the serum PSA level was 208 ng/mL. Whole-body bone scintigraphy showed no evidence of skeletal metastasis. The clinical stage was determined as cT2cN0M1. Androgen deprivation therapy (ADT) was initiated two months postoperatively. Following the initiation of ADT, the PSA level decreased progressively over time. The patient has remained progression-free 20 months after pulmonary resection.

## Discussion

The present case highlights three important issues. First, reliance on a patient’s past oncological history can lead to diagnostic anchoring and potential misdiagnosis. Although RCC has the potential to recur or metastasize even decades after initial treatment [3-5], this case demonstrates that assuming recurrence based solely on prior malignancy may be misleading. In our patient, a 24-year history of RCC initially guided the differential diagnosis, but histopathological analysis revealed a completely unrelated malignancy - metastatic prostate cancer that had previously gone undiagnosed.

Second, in patients presenting with new pulmonary nodules after a prolonged disease-free interval (DFI), the possibility of a second primary malignancy should also be considered. Particularly in older adults, the likelihood of developing an additional malignancy increases with age and time, and new lesions should not be automatically attributed to a previous cancer. A comprehensive differential diagnosis - including unrecognized neoplasms and non-malignant etiologies - is warranted.

Third, this case highlights the indispensable role of histopathological confirmation in determining the correct diagnosis and guiding appropriate treatment. In patients with suspected pulmonary metastases, especially when RCC is in the differential diagnosis, obtaining tissue safely and accurately is essential. In the present case, chest and abdominal CT revealed no lesions suggestive of metastasis or primary tumor other than the bilateral pulmonary nodules, and even if fluorodeoxyglucose (FDG) uptake had been observed in the pulmonary nodules or elsewhere on PET-CT, biopsy of the pulmonary nodules would have remained essential. Given the hypervascular nature of RCC - often retained in its metastatic sites - transbronchial or percutaneous biopsy may carry a significant risk of bleeding [6,7]. In our case, VATS provided a safe and definitive diagnostic approach. Had treatment been initiated based solely on clinical suspicion of RCC recurrence without histological confirmation, the therapeutic strategy and prognosis might have been different markedly, potentially resulting in suboptimal management. This underscores the importance of not relying exclusively on a patient's oncological history and highlights the necessity of histological confirmation to guide treatment [8]. Although recent advances in minimally invasive modalities - such as percutaneous biopsy, endobronchial ultrasound-guided transbronchial needle aspiration (EBUS-TBNA), and the guide sheath method - have significantly improved both safety and diagnostic accuracy, these approaches may still be limited in cases involving hypervascular tumors or anatomically challenging lesions [9-14]. VATS remains a valuable and justified diagnostic option, particularly when the risk of bleeding or inconclusive sampling is of concern.

In summary, this case underscores the importance of avoiding diagnostic assumptions based on a patient’s medical history and emphasizes the need for a comprehensive, histopathologically confirmed evaluation. It also illustrates that VATS can play a critical role in achieving a definitive diagnosis when other biopsy methods may be inadequate or unsafe.

## Conclusions

In patients with a history of malignancy, newly emerging pulmonary nodules after a long disease-free interval should raise suspicion not only for recurrence but also for a second primary cancer. VATS remains a safe and feasible diagnostic option, especially in patients with a prior history of RCC. A rigorous diagnostic workup, including histological confirmation, is essential to avoid inappropriate management and to ensure that patients receive the most effective intervention based on the true nature of the lesion.
